# *In vivo *monitoring of fetoplacental Vegfr2 gene activity in a murine pregnancy model using a Vegfr2-luc reporter gene and bioluminescent imaging

**DOI:** 10.1186/1477-7827-9-51

**Published:** 2011-04-16

**Authors:** Jonathan M Greene, Chad W Dunaway, Susan D Bowers, Brian J Rude, Jean M Feugang, Peter L Ryan

**Affiliations:** 1 Department of Pathobiology and Population Medicine, Mississippi State University College of Veterinary Medicine, Mississippi State, MS, USA; 2 Facility for Organismal and Cellular Imaging, Mississippi State University, Mississippi State, MS, USA; 3 Department of Animal and Dairy Sciences, Mississippi State University, Mississippi State, MS, USA

## Abstract

**Background:**

Vascular endothelial growth factor receptor-2 (VEGFR2) plays a pivotal role in angiogenesis by eliciting vascular endothelial cell growth when bound to VEGF, a powerful pro-angiogenic ligand. While Vegf and Vegfr2 are expressed throughout gestation, the latter third of gestation in mice is characterized by a marked increase in fetoplacental angiogenesis. Thus, the objective of this study was to determine the feasibility of monitoring fetoplacental Vegfr2 gene activity non-invasively using a Vegfr2-luc reporter transgenic mouse and bioluminescent imaging.

**Methods:**

Imaging parameters were optimized using two wild-type (WT) females, bearing Vegfr2-luc fetuses. Then, seven WT females, bred to Vegfr2-luc males, were imaged from gestational day (GD) 12 to 18 to determine the usefulness of the Vegfr2-luc mouse as a model for studying fetoplacental Vegfr2 activity during pregnancy. Semi-quantitative RT-PCR of Vegfr2 was also performed on whole fetoplacental units during this time. Additionally, resultant neonates were imaged at postnatal day (PND) 7, 14 and 21 to monitor Vegfr2 activity during post-natal development.

**Results:**

Fetoplacental Vegfr2 gene activity was detected as light emissions beginning on GD 12 of gestation and increased throughout the imaging period (P < 0.05), and this paralleled the Vegfr2 mRNA data obtained from RT-PCR analysis. A decline in fetoplacental light emissions was associated with a poor pregnancy outcome in one pregnancy, indicating that this approach has potential use for studies monitoring pregnancy well being. Additionally, neonatal Vegfr2 activity was detected at PND 7, 14 and 21 but declined with time (P < 0.0001).

**Conclusions:**

*In utero *fetoplacental Vegfr2 gene activity was monitored longitudinally in a quantitative manner using a luciferase reporter gene and bioluminescent imaging during the latter third of gestation. This study demonstrates the feasibility of using the Vegfr2-luc mouse to monitor late gestation fetoplacental angiogenic activity under normal and experimental conditions. Additionally, neonatal Vegfr2 gene activity was monitored for three weeks postpartum, allowing continuous monitoring of Vegfr2 activity during the latter third of gestation and postnatal development within the same animals.

## Background

Angiogenesis is the process by which new vasculature develops from preexisting vascular structures, and vascular endothelial growth factor A (VEGFA) and its cell-surface receptor, vascular endothelial growth factor receptor 2 (VEGFR2), are two proteins that are vital for this process. As a pro-angiogenic factor, VEGFA has been described as the most potent stimulator of angiogenesis while VEGFR2 is considered to be the primary receptor by which VEGFA elicits its pro-angiogenic effects, including the stimulation of endothelial cell growth in developing tissues[1,2]. While Vegf and Vegfr2 expression is a hallmark process of angiogenesis during normal wound healing and tumor development, transcription of Vegf and Vegfr2 is also critical for pregnancy success [3-9].

Recent advances in bioluminescent imaging technology have allowed real-time monitoring of gene expression *in vivo *using transgenic animal models. In these models, a light producing enzyme, such as firefly luciferase, is used as a reporter gene by incorporating it in the animal genome so that its expression is induced whenever the gene of interest is transcriptionally active. When luciferin, the substrate for luciferase, is administered, oxidation occurs releasing energy in the form of light which is proportional to the amount of gene activity. By allowing real-time measurements, these models allow gene expression to be studied within the physiological parameters of the living animal system [10]. Additionally, by reducing the need for numerous end-point measurements, which usually involve sacrificing animals to obtain tissue samples, fewer animals are needed to monitor physiological events over time in longitudinal studies [10] allowing the targeting of specific time points for further end-point analysis. In 2004, Zhang and colleagues [11] utilized a transgene comprised of a murine Vegfr2 promoter region cloned upstream from the luciferase gene to create founder FVB/N - Tg(Vegfr2-luc) - Xen mice (Vegfr2-luc), and recently, our laboratory has employed the use of this transgenic mouse model to study the activity of Vegfr2 in wound healing studies [10,12]. When Vegfr2 is transcriptionally activated, luciferase is also transcribed, allowing Vegfr2 expression activity to be monitored non-invasively and quantitatively in real-time using imaging equipment that is highly sensitive to low-emitting light.

While several studies describe the use of this mouse model to monitor Vegfr2 expression during wound healing [10-14], there is no information in the literature describing its use to monitor fetoplacental Vegfr2 activity longitudinally *in vivo*. Therefore, in this paper, we describe a murine pregnancy model, utilizing the Vegfr2-luc mouse, which allows real-time monitoring of fetoplacental Vegfr2 gene activity using bioluminescent imaging.

## Methods

### Animals

Care and use of animals utilized in this study were conducted in accordance with and under the approval of the Institutional Animal Care and Use Committee of Mississippi State University. Homozygous Vegfr2-luc males were purchased from Caliper Life Sciences, Inc. (Hopkinton, MA, USA), while wild type (WT) FVB/N females were obtained from an in-house colony derived from two vendors (The Jackson Laboratory Bar Harbor, ME, USA; Charles River Laboratories International, Inc. Wilmington, MA, USA). Males were caged individually and females in groups of four to five per cage until paired for breeding, after which bred females were housed individually. All animals were housed in an environmentally controlled room set at 22°C with a 12 h light/12 h dark cycle and allowed *ad-libitum *access to a phytoestrogen and alfalfa free diet (Purina Test Diet, Richmond, IN, USA) and water. In order to assess fetoplacental Vegfr2-luc expression, it was necessary to breed homozygous Vegfr2-luc males to WT FVB/N females so that only fetoplacental tissues would express luciferase under the control of the cloned Vegfr2 promoter.

### Optimization of imaging parameters

Due to the absence of information describing a Vegfr2-luc pregnancy model, a preliminary study was performed with two pregnant WT females, bred to Vegfr2-luc males, to determine the optimal imaging parameters. Females were checked daily following pairing with males, and the day on which a vaginal plug was observed was designated as gestational day (GD) 1. To avoid inferring with embryo implantation[15], imaging was not performed until GD 6, at which time the two females were imaged daily until GD 18 using the IVIS 100 Imaging System (Caliper Life Sciences, Inc. Hopkinton, MA, USA). Briefly, mice were anesthetized with isoflurane (1.5 to 3.0%) and injected intraperitoneally (i.p.), as recommended, in the lower left abdominal quadrant with luciferin (150 mg/kg; Caliper Life Sciences, Inc.) suspended in Dulbecco's phosphate buffered saline (15 mg/ml). Additionally, the abdominal region of the mice was shaven to reduce the effects of hair on light scattering and/or absorption. Previously, dermal wound healing studies utilizing the Vegfr2-luc mouse, reported that optimal luciferase activity for imaging purposes occurred 10 minutes after luciferase administration [10,11]; however, the time required for luciferin to traverse the maternal vasculature and arrive at the fetoplacental tissues was unknown. Therefore, mice were imaged for five minutes at 10, 15, 20, and 25 minutes post luciferin injection to determine the optimal time needed for luciferin distribution.

Results from this preliminary study indicated that optimal signal intensity was detected 20 minutes post luciferin injection but was not detected prior to GD 12 at which time, light emissions were consistently above background signal. Additionally, i.p. injections in the lower left abdominal region resulted in bruising over the injection site which may interfere with accurate signal acquisition from the fetoplacental tissues. Therefore, a subcutaneous (s.c.) luciferin injection in the dorsal neck region was chosen as an alternate site for luciferin delivery in subsequent mice given the similar luciferin distribution kinetic characteristics between both methods [16].

### Bioluminescence imaging of pregnant and neonatal mice

After imaging optimization, we proceeded to determine the usefulness of the Vegfr2-luc mouse as model for quantitative, longitudinal and real-time study of fetoplacental Vegfr2 gene activity during pregnancy. Seven WT females bred to Vegfr2-luc males were housed individually, and weights were recorded daily from GD 1 to 18 as an indicator of fetal growth. From GD 12 to 18 of pregnancy, bred females were imaged utilizing the optimized parameters determined from the preliminary study. Briefly, mice were anesthetized with isoflurane (1.5 to 3.0%), and then the abdominal region was shaven followed by administration of a luciferin (s.c.; 150 mg/kg BW) in the dorsal neck region. Twenty minutes after luciferin administration, mice were imaged for five minutes ventral side up using the IVIS 100 Imaging System while maintained under isoflurane (1.5 to 3.0%) anesthesia. Imaging terminated on GD 18 to prevent interfering with parturition which averaged 19.5 days post breeding in our colony.

In addition to the pregnant mice, pups (n = 27) from six pregnancies were imaged on post-natal day (PND) 7, 14, and 21 to evaluate Vegfr2 gene activity as a means of assessing post natal angiogenesis. Imaging parameters for neonatal mice were adapted from Zhang and colleagues [11]. Briefly, pups were anesthetized with isoflurane (1.5 to 3.0%), administered luciferin (s.c.; 150 mg/kg BW) in the dorsal neck region, and subsequently imaged ventrally for two minutes using the IVIS 100 Imaging System 10 minutes post injection.

### Image analysis

**Figure 1:**
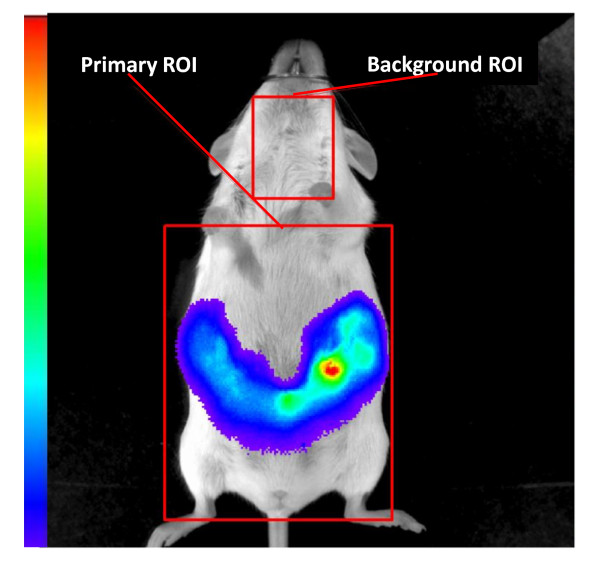
**Analysis of bioluminescent images acquired from WT females bearing Vegfr2-luc fetuses**. Images were analyzed using Living Image^® ^2.50 software. A 4.25 cm × 5.5 cm primary ROI was drawn over the abdominal region, followed by a 1.5 × 1.9 cm background ROI over the ventral neck region to correct for autoluminescence. The color scheme represents the pseudo color scale applied to the image, with the red colors indicating the greatest light emissions and the purple colors representing the least amount of light emissions.

All images were analyzed using Living Image^® ^2.50 software (Caliper Life Sciences, Inc. Hopkinton, MA, USA). Measurements were made by drawing regions of interests (ROI) on the bioluminescent images. For the pregnant females, a 4.25 cm × 5.5 cm primary ROI was drawn, covering the abdominal region of the animal being measured (Figure 1). In addition, a smaller ROI (1.5 cm × 1.9 cm) was drawn and placed on the ventral neck region to obtain a background measurement for each animal to correct for any autoluminescence that may originate from sources such as chemiluminescent metabolic processes [17]. The light emissions from the background ROI were subtracted from the light emissions from the primary ROI, and these measurements were expressed as calibrated units of photons per second (p/s). The data for the pregnant females are presented as total light emissions (p/s) and as light emissions corrected for fetoplacental mass (p/s/g), which was obtained by dividing the total light emissions for each day by the amount of weight gain relative to the initial body weight recorded on GD 1. Because the entire body of the pups expressed light, driven by the cloned Vegfr2 promoter, a background ROI could not be drawn. Measurements were corrected for the average pup weight of each litter and expressed as calibrated units of photons per second per gram (p/s/g).

### RT-PCR analysis of Vegfr2 mRNA expression

Four additional WT females were bred to Vegfr2-luc males. At each time point, GD 12, 14, 16, and 18, one female was imaged using the optimized imaging procedures to detect luciferase activity. Following imaging, females were sacrificed via cervical dislocation, and individual fetoplacental units (FPU's) were collected, imaged, and then frozen at -80°C until further analysis. Total RNA was extracted from three whole FPU's at each time point using Trizol reagent (Invitrogen Co., Carlsberg, CA, USA). RNA was quantified using a NanoDrop 1000 Spectrophotometer (Thermo Scientific, Wilmington, DE, USA) and reverse-transcribed into cDNA (RETROScript, Ambion, Austin, TX). Table 1 details the primer pairs' characteristics. The PCR conditions were as follows: 5 min at 95°C; 28 cycles of 30 sec at 95°C, 90 sec at 58°C, and 30 sec at 72°C; followed by a final extension of 10 min at 68°C. PCR products were resolved on 1.0% agarose gel stained with GelStar Nucleic Acid Stain (Lonza Walkersville Inc., Walkersville, MD, USA), and band intensity was determined using Image J 1.44c software (NIH Image). Vegfr2 gene expression data are indicated as relative expression to β-actin.

**Table 1:** Primer sequences and characteristics

Gene	GenBank Acc. #, (NCBI)	Primer sequences (5'-3')	Melt T (°C)	Amplicon size (bp)
Vegfr2	NM_010612.2	S: CTTGCAGGGGACAGCGGGAC	59.7	314
		AS: AATCGACCCTCGGCAGGGGA	59.3	
Β-actin	NM_007393.3	S: TACAATGAGCTGCGTGTGGCCC	59.7	257
		AS: AGGATGGCGTGAGGGAGAGCAT	59.5	

S, Sense; AS, Anti-sense

### Statistical analysis

One bred female was excluded from the analysis due to a failed pregnancy. Data were compared among days of gestation for the pregnant females (n = 6) and among days post-partum for pups (n = 27) using ANOVA. RT-PCR data were also analyzed using ANOVA. Least square means were calculated and separated using Fisher's least significant difference when the P-value from the ANOVA was less than 0.05. Least square means were considered to be significantly different at a value of P ≤ 0.05. Only bioluminescent imaging data from GD 12, 14, 16, and 18 were used in the final statistical analysis as there were no daily significant changes from GD 12 to GD 17. Data are presented as least square means ± standard error of the mean.

## Results and Discussion

### *In vivo *monitoring of fetoplacental Vegfr2 activity

Bioluminescent imaging of WT females, bearing Vegfr2-luc fetuses, proved to be effective for monitoring fetoplacental Vegfr2 activity during the latter third of gestation in a quantitative manner. Minimal signal was detected on GD 12 in one of the six mice, while the other five mice produced a strong signal. Beginning on GD 13 and continuing throughout the imaging period, all six mice produced a strong signal, representing fetoplacental Vegfr2 transcriptional activity. The described approach is one that utilizes a WT female bred to a Vegfr2-luc male. In contrast, utilizing a Vegfr2-luc female bred to a Vegfr2-luc male would not allow fetoplacental light emissions to be discriminated from light emissions originating from maternal tissues, such as the uterus, which expresses Vegfr2 during gestation [18] or ovarian angiogenesis from vascularized corpus lutea [19]. The advantage of utilizing a WT female is that background signal originating from maternal tissues is eliminated. This allows fetoplacental light emissions to be monitored, with large signal to background ratios, since the only tissues bearing the Vegfr2-luc transgene are those of fetal origin.

Previous studies utilizing the Vegfr2-luc mouse administered luciferin with an i.p. injection given in the animal's lower left abdominal quadrant [10-12]; however, in our preliminary work, we observed that this method of injection caused bruising at the injection site. This injection method was abandoned because the pooling of blood at the bruising site may interfere with the transmission of light through the tissue [20]. Additionally, since the uterus was rapidly expanding during this time, penetrating the uterus with an i.p. injection became a concern. Given these considerations, it was determined that an s.c injection in the dorsal neck region may be more appropriate for pregnant mouse model studies. Subcutaneous injection proved to be a reliable and repeatable method for luciferin administration, consistent with previous studies which determined that s.c. luciferin administration avoids potential misadministration associated with i.p. luciferin administration while providing similar light emission yields and luciferin distribution kinetics [16].

Preliminary observations also revealed that 20 minutes post luciferin injection yielded optimal signal intensity for imaging purposes. Wound healing studies reported optimal luciferase activity 10 minutes after luciferin administration when using the Vegfr2-luc mouse [10,11]; however, in our preliminary work, waiting ten minutes post-luciferin administration did not provide adequate time for luciferin to disperse through the fetoplacental tissues. It is likely that additional time is needed for luciferin to traverse the maternal vasculature and be delivered to the fetoplacental tissues as compared to the time needed to deliver luciferin to dorsal, dermal skin wounds.

**Figure 2:**
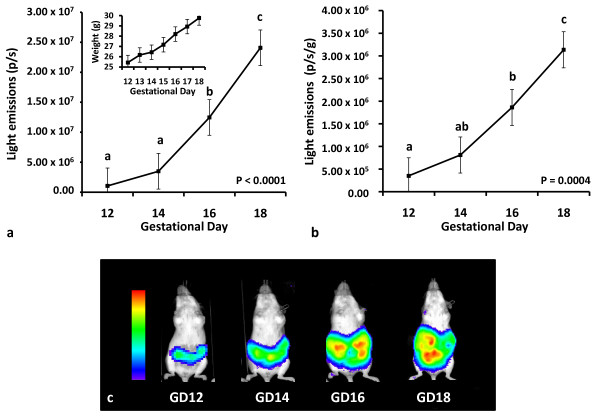
***In vivo *bioluminescent imaging of fetoplacental Vegfr2 activity**. Quantitative differences in light emissions (p/s) were found during the seven day imaging period (P < 0.05). (**a**) Comparing gestational day (GD) 12 to 18, total light emissions (p/s) increased by approximately 23-fold (P = 0.0006). The insert illustrates the increase in maternal body weight (P = 0.0008) occurring during this period, indicating fetoplacental growth. (**b**) Light emissions corrected for fetoplacental mass (p/s/g) also increased by approximately 9-fold (P < 0.0001) during this time. (**c**) Additionally, the images demonstrate qualitative monitoring of fetoplacental Vegfr2 gene activity in a complete set of images chosen from a representative animal. ^**a,b,c **^means without a common superscript differ (P ≤ 0.05).

**Figure 3:**
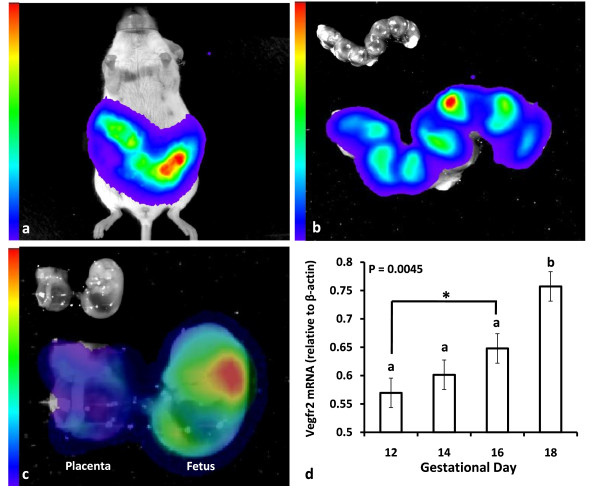
***In vivo *and *ex vivo *bioluminescent imaging and RT-PCR analysis of Vegfr2 in fetoplacental tissues**. Figures represent bioluminescent imaging of fetoplacental Vegfr2 activity (**a**) *in vivo *in a pregnant mouse, (**b**) *ex vivo *in a gravid uterus (inset bright field image), and (**c**) *ex vivo *in a whole FPU (inset bright field image) from a single pregnancy at gestational day (GD) 16. Additionally, (**d**) RT-PCR analysis revealed that Vegfr2 mRNA was present and increased in the whole FPU during GD 12 to 18. ^**a,b,c **^means without a common superscript differ (P ≤ 0.05). * means tended to differ (P < 0.10)

Utilizing the optimized imaging parameters from the preliminary study, quantitative differences in total light emissions were found during the imaging period (Figure 2a; P < 0.0001). Total light emissions on GD 12 and 14 did not differ (P > 0.05) but were less (P < 0.05) than the amount of light emissions detected on GD 16 and 18. Additionally, total light emissions on GD 18 were significantly greater (P < 0.05) than light emissions on GD 12, 14, and 16. Accounting for fetoplacental mass, quantitative differences in light emissions were also found (Figure 2b; P = 0.0004). There were no differences (P > 0.05) in light emissions between GD 12 and 14 or between GD 14 and 16. However, light emissions on GD 16 were greater (P < 0.05) than light emissions on GD 12, and light emissions on GD 18 were greater (P < 0.05) than light emissions on GD 12, 14, and 16, indicating that fetoplacental Vegfr2 gene activity was greatest on GD 18. In addition to the quantitative data, qualitative data (Figure 2c) also depicts an increase in light emissions occurring during this time period. Moreover, *in vivo *imaging of the pregnant dam (Figure 3a), followed by *ex vivo *imaging of the gravid uterus (Figure 3b) and individual FPU (Figure 3c) further revealed that the light emissions were originating from fetoplacental tissues.

Detection of light emissions during the latter third of gestation indicated that Vegfr2 was transcriptionally active in fetoplacental tissues during this timeframe. It has been previously reported that light emissions were associated with Vegfr2 expression and angiogenesis when the Vegfr2-luc mouse was used to study dermal wound healing [10,11], and indeed, semi-quantitative RT-PCR analysis, in the current study, confirmed that Vegfr2 mRNA was present in fetoplacental tissues and increased relative to β-actin (P < 0.05; Figure 3d). This is consistent with the bioluminescent data and other reports detailing an increase in Vegf mRNA during this time [21]. Moreover, end-point measurements have revealed that Vegfr2 is expressed as early as GD 7 during murine pregnancy and is continually expressed throughout gestation in fetal and placental tissues [4,22,23]. The current data indicate the ability to monitor fetoplacental Vegfr2 activity in real time and over time *in vivo *during the latter third of gestation using a luciferase reporter gene and bioluminescent imaging technology. Quantitative differences in Vegfr2 activity were detected which further suggests the usefulness of this approach for obtaining meaningful data regarding changes in fetoplacental Vegfr2 activity under experimental conditions.

Previous reports have revealed that the latter third of gestation in mice is characterized by a marked increase in the amount of fetoplacental vasculature and fetal growth [24-26]. In the current study comparing GD 12 to GD 18, there was a 23-fold increase (P < 0.0001) and 9-fold increase (P < 0.0001) in total light emissions and light emissions corrected for fetoplacental mass, respectively, indicating a large increase in fetoplacental Vegfr2 activity with the advancement of gestation. Expression of Vegfr2 is induced by increased activity of VEGF [27], a powerful pro-angiogenic factor, suggesting that the increase in fetoplacental light emissions observed in the current study represents the angiogenic activity associated with the tremendous growth of the fetoplacental vasculature network and fetus [24-26] during this period. By monitoring the transcriptional activity of Vegfr2 using the luciferase reporter gene and bioluminescent imaging, the current approach affords the capability to study late gestation fetoplacental angiogenesis from a molecular standpoint in a non-invasive, quantitative, and longitudinal manner *in vivo*. Moreover, the results are in agreement with previous work demonstrating the marked increase in angiogenic activity occurring during the latter third of gestation in mice [24,25], suggesting the usefulness of this approach for monitoring fetoplacental angiogenic activity during this period.

**Figure 4:**
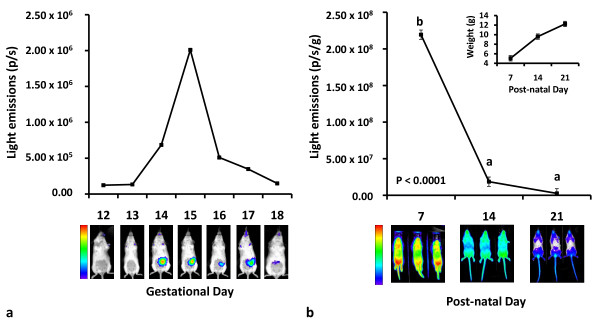
**Bioluminescent imaging of Vegfr2 activity in failed pregnancy and Vegfr2-luc neonates**. (**a**) Minimal signal was detected on gestational day (GD) 12 and 13. Light emission increased on GD 14 and 15; however, light emission drastically declined on GD 16, 17, and 18. This decrease in light emissions was associated with a poor pregnancy outcome, in which there were no surviving offspring. (**b**) Light emissions were detected in the neonates, and from PND 7 to 21, there was an 83-fold reduction in light emissions (P < 0.0001), indicating a decrease in Vegfr2 gene expression as the neonates progressed in development. The inserted graph depicts the increase in average pup weight (P < 0.0001) occurring during this period as an indicator of growth. The images are a representative group of animals imaged during this period. ^**a,b,c **^means without a common superscript differ (P ≤ 0.05).

We also report a serendipitous, yet interesting, finding in which a dramatic decline in light emissions was associated with a poor outcome in a seventh pregnancy (Figure 4a). During the imaging period for one mouse, light emissions remained minimal and similar to background measurements on GD 12 and 13, indicating a weak signal. Light emissions increased above background measurements on GD 14 and 15. However on GD 16, there was a four-fold decrease in light emissions, followed by a continuing decline on GD 17 and 18 of gestation. Interestingly, the decline in light emissions was associated with a poor pregnancy outcome in which there were no surviving offspring. Previous studies have revealed that VEGFR2 and VEGF are vital for fetal development and survival [3,5,9]. Moreover, Ferrara and colleagues speculated that if Vegf activity during pregnancy decreases to a critical point, organogenesis may be permanently impaired [5]. This may suggest that the observed decline in Vegfr2 gene activity in the current study was associated with a compromised pregnancy that resulted in fetal demise, or the decline in light emissions could be simply the result of fetal death rather than fetal death stemming from an angiogenic defect. Either way, this unexpected observation further highlights the importance of VEGFR2 during pregnancy and demonstrates that the current approach could be utilized to evaluate pregnancy loss resulting from inactivation of angiogenic pathways.

The approach detailed in the current study offers a novel method for studying late gestational angiogenesis in mice. However, anesthetizing pregnant mice poses some concern for the developing fetuses. While isofluorane exposure has been reported to have a deleterious effect on early mouse embryo development [28], recent reports have demonstrated that fetal exposure to isofluorane during ultrasonography on GD 8.5 or GD 10.5 has no significant adverse biological effects [29], which is consistent with the lack of negative effects observed in the current study. Given that the model described herein requires the use of isofluorane during late gestation, perhaps the deleterious effects that isofluorane has on early mouse embryo development were avoided. However, it cannot be fully determined whether isofluorane anesthesia contributed to the pregnancy demise in the seventh mouse.

### *In vivo *monitoring of neonatal Vegfr2 activity

Angiogenesis is a developmental process that becomes relatively quiescent in adulthood except during certain conditions such as tumor development [30], wound healing [31] and corpus luteum development [19]. However, angiogenesis, and thus Vegf and Vegfr2 activity, are extremely important during the neonatal period with activity declining with age [21,32]. Neonatal mice (n = 27) from six pregnancies were imaged at PND 7, 14 and 21 to monitor Vegfr2 gene activity during postnatal development. Bioluminescent signal was detected at PND 7, 14 and 21 and differed over the imaging period (P < 0.0001; Figure 4b). From PND 7 to 14, there was a 11.7-fold decrease in light emissions (P < 0.0001), followed by a tendency for a 7-fold decrease in light emissions from PND 14 to 21 (P = 0.08). Overall, there was an 83-fold reduction in light emissions from PND 7 to 21 (P < 0.0001), indicating a decrease in Vegfr2 gene expression and, thus, angiogenesis as the neonates progressed in development. While the decline of Vegfr2 activity during the neonatal period is not a novel observation [11,21], the approach described in this study offers continuous monitoring of Vegfr2 activity during the latter third of gestation and subsequent neonatal development within the same animals.

## Conclusions

In the current study, the usefulness of the Vegfr2-luc mouse as a research model to measure fetoplacental Vegfr2 gene activity *in vivo *was assessed and determined to be effective using bioluminescent imaging. Light emissions, representative of fetoplacental Vegfr2 gene activity, were detected on GD 12 to 18, monitored quantitatively and longitudinally during this period, and were consistent with traditional end-point molecular analysis. This novel approach offers the ability to obtain meaningful data depicting changes in fetoplacental Vegfr2 activity *in utero*. Neonatal Vegfr2 activity was also monitored in the resultant offspring during the first three weeks of life. Accordingly, this approach may have great potential for studying developmental perturbations encountered *in utero *and how they may influence late fetal and early postnatal angiogenesis.

## Competing interests

The authors declare that they have no competing interests.

## Authors' contributions

JG conceived the study and participated in the design of the study. JG performed all experiments, conducted the statistical analysis, interpreted the data and drafted the manuscript. PR conceived the study, participated in the design of the study and assisted with data interpretation as well as helped to draft the manuscript. JF participated in the imaging procedures and molecular analysis and assisted with data interpretation. CD and SB aided with the image acquisition and data interpretation. BR participated in the study design. All authors read and approved the final manuscript.
